# Complete mitochondrial genome data and phylogenetic analysis of the Great Marquis, *Bassarona dunya* (Doubleday, 1848) (Lepidoptera: Nymphalidae: Limenitidinae) from Malaysia

**DOI:** 10.1080/23802359.2023.2167476

**Published:** 2023-01-26

**Authors:** Marylin Miga, Yong Zi Yap, Puteri Nur Syahzanani Jahari, Sivachandran Parimannan, Heera Rajandas, Muhammad Abu Bakar-Latiff, Yap Jing Wei, Mohd Shahir Shamsir, Faezah Mohd Salleh

**Affiliations:** a Department of Biosciences, Faculty of Science, Universiti Teknologi Malaysia, Johor Bahru, Malaysia; b Faculty of Applied Sciences, Centre of Excellence for Omics-Driven Computational Biodiscovery (COMBio), AIMST University, Bedong, Malaysia; c Faculty of Applied Sciences and Technology (FAST), Environmental Management and Conservation Research Unit (ENCORE), Universiti Tun Hussein Onn Malaysia, Pagoh Higher Education Hub, Muar, Malaysia; d Faculty of Applied Sciences and Technology (FAST), Centre of Research for Sustainable Uses of Natural Resources (SUNR), Universiti Tun Hussein Onn Malaysia, Pagoh Higher Education Hub, Muar, Malaysia

**Keywords:** The Great Marquis, *Bassarona dunya*, complete mitogenome, Limenitidinae, Malaysia

## Abstract

The Great Marquis or *Bassarona dunya* is a butterfly species commonly found in the tropical regions of Asia, America, and Africa. This butterfly is a member of the subfamily Limenitidinae and the classification within this subfamily has been unstable. Here, we report the first complete mitochondrial genome (mitogenome) of *B. dunya* sampled from Malaysia. The mitogenome is 15,242 bp long, comprising a set of 13 protein-coding genes (PCGs), 22 transfer RNAs (tRNAs), two ribosomal RNAs (rRNAs), and an A + T rich region. All PCGs were initiated by the typical ATN codon, except for COX1 which started with a CGA start codon. Nine PCGs were terminated with a TAA or TAG stop codon, while COX1, COX2, NAD4, and NAD5 ended with an incomplete T. The 12S and 16S rRNAs were 716 bp and 1269 bp in length, respectively. Phylogenetic analysis supported the placement of *B. dunya* within Limenitidinae with a high support value.

## Introduction

**Figure 1.**
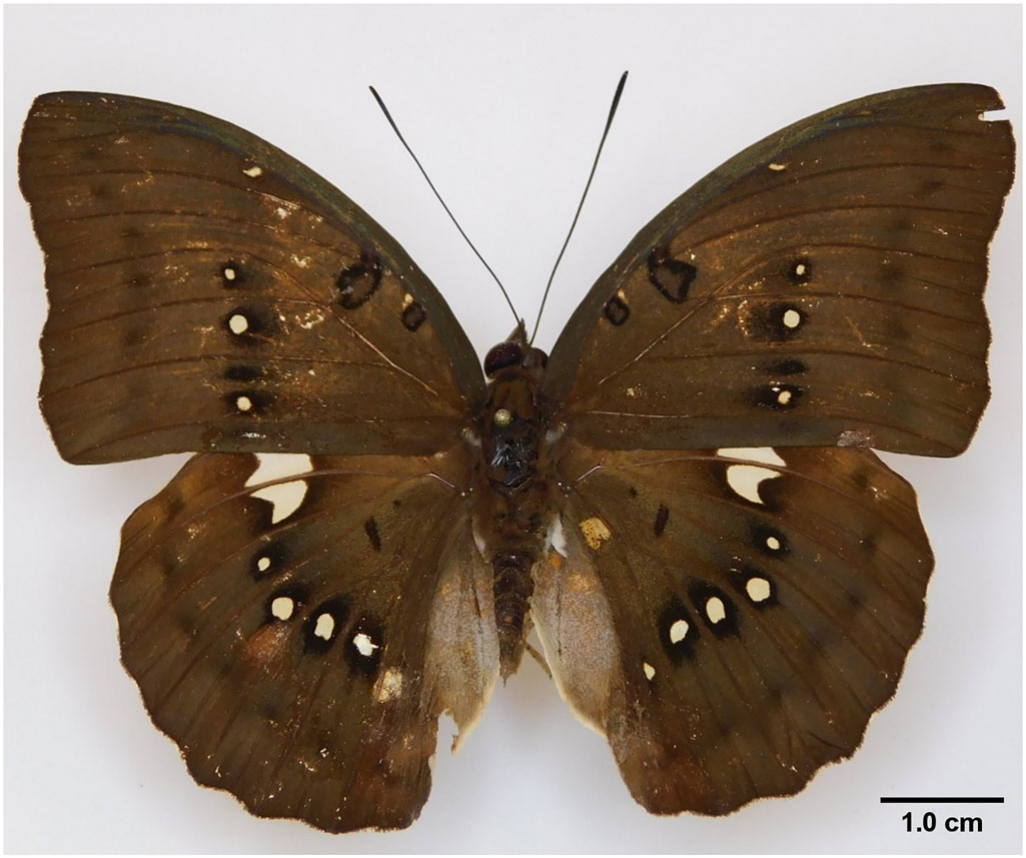
A reference image of the sequenced *Bassarona dunya* collected from Ayer Hitam Forest Reserve, Johor, Malaysia.

*Bassarona dunya* (Doubleday, 1848) is a butterfly from the subfamily Limenitidinae and is distributed mainly in the tropical regions of Asia, America, and Africa (Hui-Yun et al. 2022). They are commonly identified based on the uniform pale brown, with a postdiscal series of creamy white spots on both wings (Corbet et al. 2021) (Figure 1). This subfamily has been subjected to a long history of complex taxonomic classification, and recent phylogenetic studies based on molecular data have gradually unraveled their taxonomic position both at the subfamily and lower classification level (Dhungel and Wahlberg 2018; Wu et al. 2019; Wahlberg et al. 2020; Hui-Yun et al. 2022; Liu et al. 2022). To date, a complete mitogenome of *B. dunya* has yet to be reported publicly. In this study, we sequenced and analyzed the first complete mitogenome of *B. dunya* from Malaysia which will become an important resource in further addressing the taxonomic issues of this subfamily.

## Materials and methods

**Figure 2.**
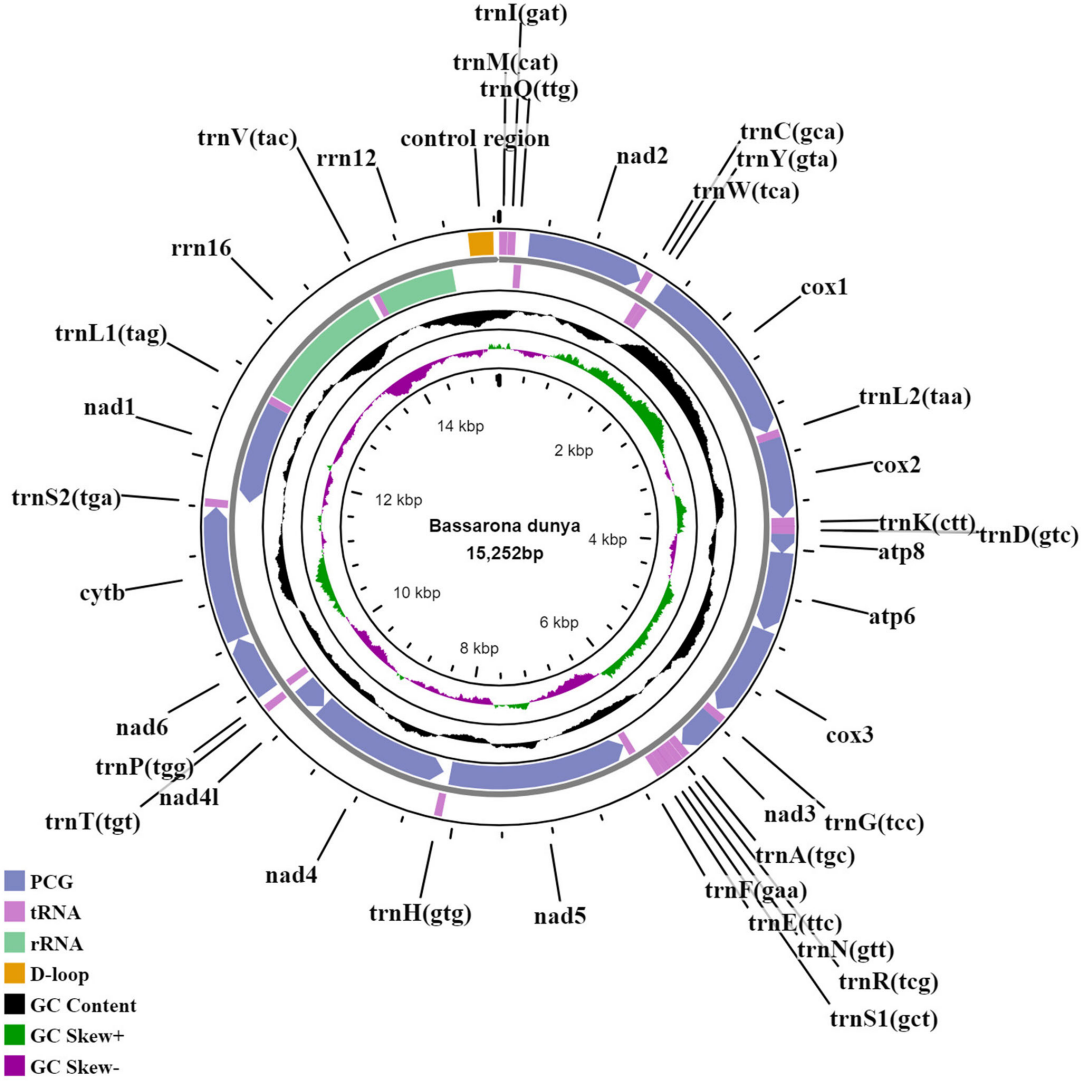
Circular mitogenome map of *B. dunya* generated in this study.

The adult *B. dunya* was collected from Ayer Hitam Forest Reserve Johor, Malaysia (2.025680, 102.794151). The specimen was deposited in Universiti Tun Hussein Onn Malaysia (UTHM) (https://uthm.edu.my; Dr Aqilah Awg Abdul Rahman; aqilah@uthm.edu.my) with the voucher ID DIB031. Total genomic DNA was extracted from the hind leg tissue using the Qiagen Blood and Tissue Kit (Qiagen, Valencia, CA) following the manufacturer’s instruction, and fragmented via a Bioruptor^®^ system. For library preparation, NEBNext^®^ Ultra™ II DNA Library Prep Kit for Illumina^®^ was used prior to sequencing by Illumina NovaSeq 6000 system (PE150). The raw reads obtained were pre-processed using FastQC (https://www.bioinformatics.babraham.ac.uk/projects/fastqc/), and then trimmed for sequencing adapters, as well as low quality reads using AdapterRemoval (Schubert et al. 2016). For the mitogenome assembly, NOVOPlasty v.4.2 (Dierckxsens et al. 2017) was used, with a seed reference (BOLD ID: YB-KHC6757) before running through a PALEOMIX BAM pipeline (Schubert et al. 2014) (default parameters) to remove reads shorter than 15 bp after trimming (Miga et al. 2022). A total of 10,447,566 reads were retained and annotated using MITOS2 web server (Bernt et al. 2013). The predicted protein-coding genes (PCGs) were further verified via ORFFinder (https://www.ncbi.nlm.nih.gov/orffinder/) to improve the annotation. Tablet (Milne et al. 2010) was also used to visualize the assembled mitogenome for indels and sequence coverage. A mitogenome map of the sequenced *B. dunya* in this work was generated using Proksee (https://proksee.ca/), an updated version of the CGView web server (Grant and Stothard 2008) as displayed in Figure 2.

**Figure 3.**
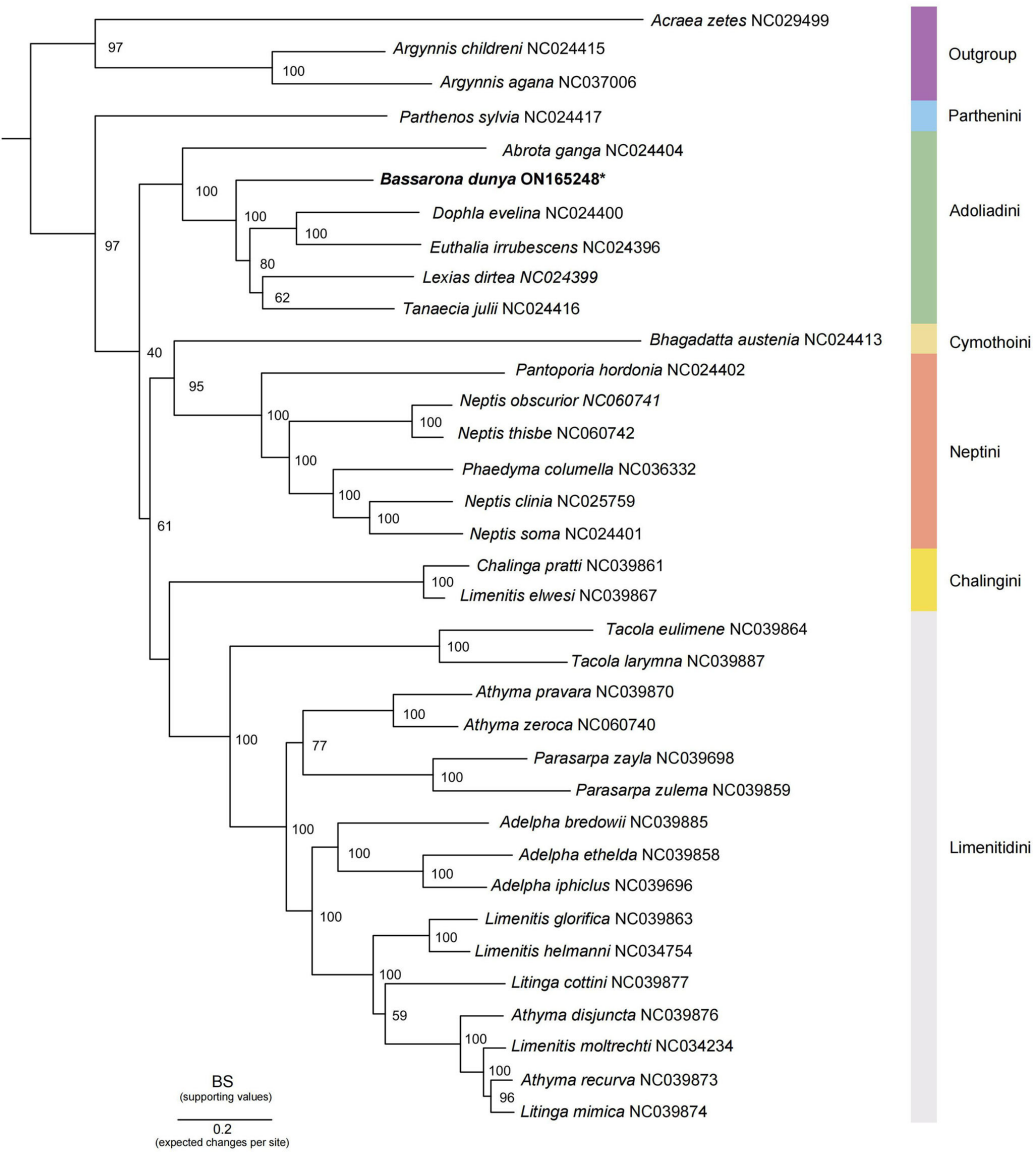
Phylogenetic analysis of *B. dunya* sequenced in this study, as indicated with an asterisk, and other 34 Nymphalidae mitogenomes. The node values represent the bootstrap of ultrafast 5000 replicates (BS), whereas each color indicates the tribes in Limenitidinae. Outgroup: *Acraea zetes* (NC029499) (Timmermans et al. 2016); *Argynnis sagana* (Liu et al. 2018); *Argynnis childreni* (NC024415) (Wu et al. 2014). Ingroup: *Abrota ganga* (NC024404), *Euthalia irrubescens* (NC024396), *Lexias dirtea* (NC024399), *Dophla evelina* (NC024400), *Tanaecia julii* (NC024416), *Bhagadatta austenia* (NC024413), *Parthenos sylvia* (NC024417), *Neptis soma* (NC024401), and *Pantoporia hordonia* (NC024402) (Wu et al. 2014); *Adelpha bredowii* (NC039885), *Adelpha ethelda* (NC039858), *Adelpha iphiclus* (NC039696), *Athyma pravara* (NC039870), *Athyma recurva* (NC039873), *Athyma disjuncta* (NC039876), *Limenitis glorifica* (NC039863), *Litinga mimica* (NC039874), *Litinga cottini* (NC039877), *Parasarpa zulema* (NC039859), *Parasarpa zayla* (NC039698), *Tacola larymna* (NC039887), *Tacola eulimene* (NC039864), *Chalinga pratti* (NC039861), and *Limenitis elwesi* (NC039867) (Wu et al. 2019); *Athyma zeroca* (NC060740), *Neptis obscurior* (NC060741), and *Neptis thisbe* (NC060742) (Liu et al. 2021); *Neptis clinia* (NC025759) (Tang, et al. 2014); *Phaedyma columella* (NC036332) (Chen et al. 2018); unpublished: *Limenitis helmanni* (NC034754) and *Limenitis moltrechti* (NC034234); this study: *Bassarona dunya* (ON165248).

To investigate the phylogenetic relationships within this subfamily, 13 concatenated PCGs from 35 Nymphalidae mitogenome sequences were used to reconstruct the maximum-likelihood (ML) tree. As we focus on the subfamily Limenitidinae, the combined dataset comprises three Heliconiinae and 32 Limenitidinae, including four recognized tribes (Dhungel and Wahlberg 2018). The mitogenomes of *Acraea zetes* (NC029499), *Argynnis sagana* (NC037006), and *Argynnis childreni* (NC024415) from the subfamily Heliconiinae were used as the outgroups. Phylogenetic analysis was performed using IQ-Tree (Nguyen et al. 2015), under an edge-linked partition model with 5000 ultrafast bootstrap replicates. The best partitioning schemes and evolutionary model were determined by PartitionFinder v2.2.1 (Lanfear et al. 2017) (Figure 3).

## Results

The complete mitogenome of *B. dunya* (GenBank accession no.: ON165248) is 15,242 bp in length, encoding 13 PCGs, 22 transfer RNAs (tRNAs), two ribosomal RNAs (rRNAs), and a control region. The mitogenome has a depth coverage of 167X and is A + T biased (81.45%). It has a nucleotide composition of A (38.51%), T (42.93%), TC (10.96%), and G (7.59%). The PCGs have a total length of 11,226 bp, whereas the tRNAs are 1461 bp, ranging from 61 bp (tRNA-Ser) to 71 bp (tRNA-Lys). The length of the 12S and 16S rRNA genes are 716 bp and 1269 bp, respectively. Out of the 13 PCGs, 12 were initiated by the typical ATN start codon, while COX1 utilizes the CGA codon. Four PCGs (COX1, COX2, NAD4, and NAD5) ended with an incomplete stop codon, while others were terminated either by TAA or TAG stop codon. Additionally, BLASTn analysis (McGinnis and Madden 2004) was done on the COX1 sequence of this species which showed 97.04% similar to the *B. dunya* COX1 sequence in GenBank (accession no.: GQ864742).

Phylogenetic analysis showed that all tribes formed monophyletic clades. The ML tree recovered the relationship of Parthenini+(Adoliadini+((Cymothoini + Neptini)+(Chalingini + Limetidini))). The analysis also showed that the newly sequenced *B. dunya* formed a clade with the other Asian Adoliadini genera (Dhungel and Wahlberg 2018; Hui-Yun et al. 2022) and is sister to *Abrota ganga* (NC024404) (Wu et al. 2014). The placement of *B. dunya* within the clade is also highly supported (100%).

## Discussion and conclusions

This study provided the complete mitogenome of *B. dunya* and was deposited in GenBank (accession no.: ON165248). The newly sequenced *B. dunya* reported shared similar gene characteristics with the other genera in Limenitidinae. It has the shortest mitogenome length compared to other genera in the Adoliadini tribe, but it is still within the range of the typical Lepidoptera mitogenome. These differences could be influenced by the length of the control region (Cheng et al. 2018). The utilization of the CGA start codon is also common in Lepidoptera mitogenome and the phenomena of the incomplete stop codon is presumed to be associated with the polyadenylation processes (Chen et al. 2020). For phylogenetic analysis, the monophyly of Adoliadini is strongly supported (BS = 100), and the position of Parthenini as sister to the other Limenitidinae tribes is consistent with other reported studies (Wahlberg et al. 2020; Hui-Yun et al. 2022; Liu et al. 2022). The mitogenome of *B. dunya* sequenced in this study provides useful information on the phylogeny of the Adoliadini tribe, and to further address their taxonomic issues, extensive taxon sampling should be considered.

## Data Availability

The genome sequence data that support the findings of this study are openly available in GenBank of NCBI at https://www.ncbi.nlm.nih.gov/ under the accession no. ON165248. The associated BioProject, SRA, and BioSample numbers are PRJNA753627, SRR15422669, and SAMN20720549, respectively.
